# Long non-coding RNA OIP5-AS1 regulates smoke-related chronic obstructive pulmonary disease via targeting micro RNA -410-3p/IL-13

**DOI:** 10.1080/21655979.2021.2000199

**Published:** 2021-12-07

**Authors:** Wenbo Hao, Fei Lin, Hanbing Shi, Zhanjiang Guan, Yunfei Jiang

**Affiliations:** a Cardiothoracic Surgery, The Third Affiliated Hospital of Qiqihar Medical University, Qiqihar, Heilongjiang, China; b Endocrinology Department, The Third Affiliated Hospital of Qiqihar Medical University, Qiqihar, Heilongjiang, China; c Department of Respiratory and Critical Care Medicine, The Third Affiliated Hospital of Qiqihar Medical University, Qiqihar, Heilongjiang, China; d Department of Critical Care Medicine, The Third Affiliated Hospital of Qiqihar Medical University, Qiqihar, Heilongjiang, China

**Keywords:** OIP5-AS1, miR-410-3p, IL-13, biomarker, inflammation

## Abstract

This investigation aimed to assess the levels of serum OIP5-AS1 and micro RNA-410-3p (miR-410-3p) in patients with chronic obstructive pulmonary disease (COPD) and their potential molecular mechanism. The levels of OIP5-AS1 and miR-410-3p as well as mRNA levels of IL-13 were measured. Pearson variable linear test was applied to analyze the correlations between forced expiratory volume in 1 second (FEV1) and OIP5-AS1. The receiver operating characteristic curve was used to predict the predictive possibility of OIP5-AS1. The viable cells were detected by 3-(4,5-dimethylthiazol-2-yl)-2,5-diphenyltetrazolium bromide (MTT) and flow cytometry was used to detect the cell apoptosis. An enzyme-linked immunosorbent assay was performed to indicate the inflammatory situation of 16HBE cells. Luciferase activity assay was conducted to examine the relationships between OIP5-AS1 and miR-410-3p together with miR-410-3p and IL-13. Augmented levels of OIP5-AS1, declined levels of miR-410-3p, and enhanced expression of IL-13 were unveiled. The expression of OIP5-AS1 and miR-410-3p was related to the ratio of FEV1 respectively. OIP5-AS1 might serve as a diagnostic biomarker. Interference of OIP5-AS1 restored the abnormal cell viability, apoptosis, and inflammation in cigarette smoke extract (CSE)-stimulated 16HBE cells by regulating miR-410-3p and IL-13. OIP5-AS1 appeared to be a biomarker for distinguishing COPD patients from smokers. OIP5-AS1/miR-410-3p/IL-13 exerted function on the cell viability, apoptosis, and inflammation in CSE-steered cell models.

## Introduction

As a treatable disease, chronic obstructive pulmonary disease (COPD) is a preventable lung inflammation [1]. The etiology of COPD is complex and diverse, and the disease is the result of environmental (cigarette smoke, kitchen combustion products, and heating coal products, etc.) and genetic interaction [2,3]. COPD is usually accompanied by clinical symptoms such as progressive dyspnea [4]. COPD elicits a systemic immune response and leads to the loss of lung function [5]. However, there is still no great progress in the early diagnosis and management of COPD. Over 3 million cases were died due to COPD in 2017 [6]. COPD is one main reason that leads to mortality and complications [7]. Thus, COPD is a serious threat to human health and has become a public health burden to be solved urgently [8].

Recently, abnormal expression levels of lncRNAs in lung tissue of COPD have been investigated, which indicates that long non-coding RNA (lncRNA) may be connected to the occurrence and development of COPD [9]. In patients with COPD, the expression of lncRNA Micro RNA-15 (miR-15) is enhanced and it can regulate cell apoptosis and inflammation by inhibiting miR-218-5p [10]. Besides, Hu et al. provide that MALAT1 is highly expressed in the lung tissue specimens of COPD patients and it may serve as a biomarker for diagnosing and treating COPD patients [11]. Many investigations on the roles of OIP5-AS1 have been performed these years. In a publication of 2020, OIP5-AS1 suppresses miR-29a, which attenuates cell apoptosis during myocardial ischemia/reperfusion [12]. In lung adenocarcinoma, the expression of OIP5-AS1 is at a high level in lung tissues and it may be an oncogene by promoting cell proliferation by regulating miR-448/Bcl-2 [13]. More importantly, in bronchial asthma, the levels of OIP5-AS1 are elevated in the BEAS‑2B cells managed with Der p1 [14]. These elucidate that OIP5-AS1 may be linked to the regulation of airway diseases.

Therefore, this study aimed to identify the expression of OIP5-AS1 in COPD patients and provided its diagnostic possibility. Furthermore, the functions of OIP5-AS1 on viable cells, apoptotic cells, and inflammatory situations were evaluated in the bronchial epithelial cell 16HBE treated with cigarette smoke extract (CSE). The potential target gene and pathway of OIP5-AS1 in regulating COPD were also revealed.

## Materials and methods

### Patients and sample collection

All participants were informed of our study plan and signed the written informed consent forms. This observation was approved by the Ethics Committee of The Third Affiliated Hospital of Qiqihar Medical University (Ethical number: 2,018,103) and all procedures conformed to the relevant rules and guidelines. Collection of study samples and clinical information were performed strictly in accordance with the regulations. A total of 62 COPD patients, 59 smokers, and 55 normal controls were contained in this investigation. The reference standard of COPD patients was the global initiative on COPD, namely, the ratio of forced expiratory volume in 1 second (FEV1) to forced vital capacity (FVC) in the first second of pulmonary function after inhalation of bronchodilator was less than 0.7 and in combination with clinical symptoms and signs [15]. Irreversible obstructive pulmonary ventilation dysfunction caused by other factors was excluded. The volunteers in the normal control group were recruited from The Third Affiliated Hospital of Qiqihar Medical University in the same period and matched with the basic information of patients with COPD. The exclusion criteria were the diagnosis of asthma, connective tissue disease, other types of obstructive pulmonary disease, malignant tumor, etc. About 10 mL venous blood was collected from all enrolled subjects and relevant clinical data were collected.

### CSE preparation

CSE was prepared according to the previously reported method [16]. In short, a cigarette burns with a modified apparatus with a driving device. The smoke was dissolved into PBS solution and filtered with a 0.2 μm filter. The CSE stock solution (PH = 7.0) was a solution with an optical density of 0.43 ± 0.02 at 320 nm. 100% CSE samples were serially dissolved with PBS to working concentrations of 0.5, 1, 2, and 4%.

### Cell model establishment and transfection

Human bronchial epithelial cell 16HBE was commonly used in the studies of COPD [17,18]. 16HBE cells were purchased from Procell (Wuhan, China) and fostered in the RPMI-1640 culture with 10% FBS and 1% P/S. The small interference OIP5-AS1 (si-OIP5-AS1) and siRNA negative control (si-NC), together with miR-410-3p inhibitor sequences, miR-410-3p mimic sequences, and their negative control (miR-NC) were all obtained from GenePharma (Shanghai, China). The sequences were as follows: si-OIP5-AS1, 5ʹ-GGCTTTGTGTTCCTTATCACAGG-3ʹ; si-NC, 5ʹ-TTCTCCGAACGTGTCACGTTT-3ʹ; miR-410-3p mimic, 5ʹ-AAUAUAACACAGAUGGCCUGU-3ʹ; miR-410-3p inhibitor, 5ʹ-ACAGGCCAUCUGUGUUAUAUU-3ʹ and miR-NC 5ʹ-CAGUACUUUUGUGUAGUACAA-3ʹ.

The transfection experiments were used Lipofectamine 3000 and carried out based on the recommended protocols [19]. The cells with 70–90% confluent were seeded into a 6-well plate. The target sequences were diluted with opti-MEM medium separately. Then, the two solutions prepared before were mixed and added to cells. After 48 h, all transfected cells were harvested for qRT-PCR and other assays.

### Quantitative real-time PCR (qRT-PCR)

The total RNA samples in blood samples were gathered by TRIzol reagent [20]. For OIP5-AS1, cDNA was reversed with the help of the 1st strand cDNA synthesis kit (Biomarker, Beijing, China). For miR-410-3p, the cDNA was obtained by using miRNA cDNA synthesis kit (Vazyme, Nanjing, China). The 2X SYBR Green Fast qPCR mix (Biomarker, Beijing, China) was applied to examine the expression levels of OIP5-AS1. MiRNA fluorescent quantitative PCR detection kit (Baiaobolai, Beijing, China) was used for miR-410-3p detection. Along with this, as reference genes, the expression of U6 and GAPDH was also assessed. The primers of OIP5-AS1, GAPDH, miR-410-3p, and U6 were designed and synthsized by BGI (Shenzhen, China). The primer sequences were as follows: OIP5-AS1 forward, 5ʹ-TGCGAAGATGGCGCAGTAAG-3ʹ and reverse, 5ʹ-TAGTTCCTCTCGTCTGGCCG-3ʹ; GAPDH forward, 5ʹ-AGGTGAAGGTCGGAGTCAACG-3ʹ and reverse: 5ʹ-AGGGGTCATTGATGGCAACA-3ʹ; miR-410-3p forward, 5ʹ-GTCAGCGCAATATAACACAG-3ʹ and reverse, 5ʹ-GAGAACAGCTCTGTGTTATAT-3ʹ; U6 forward, 5ʹ-CTCGCTTCGGCAGCACA-3ʹ; and reverse, 5ʹ-AACGCTTCACGAATTTGCGT-3ʹ; and IL-13 forward, 5ʹ-ACCCAGAGGATATTGCATGG-3ʹ, and reverse 5ʹ-TGGGCTACTTCGATTTTGGT-3ʹ. Each reaction was carried out three times to eliminate the error.

### 3-(4,5-dimethylthiazol-2-yl)-2,5-diphenyltetrazolium bromide (MTT) assay

All the procedures were in line with the specification of MTT reagent (Beyotime, Shanghai, China) [21]. The transfected cells were collected and a cell suspension with an appropriate concentration was prepared. The cell suspension was inoculated into a 96-well plate at a cell concentration of 2 × 10^3^ per well. After the culture was continued for 48 h, 10 µl MTT was added to each well and the wells were incubated for a further 2 h. The A490 values in each well were detected by the microplate reader.

### Detection of apoptotic cell rate

When the cells in the 6-well plate grew to 70%, the cells were digested with trypsin. Then cells were collected and mixed with Annexin V-FITC and propidium iodide solution evenly. After incubation in the dark for 10 min at room temperature, flow cytometry was used for detection. All these procedures were performed based on the protocols of the TACS Annexin V-FITC Apoptosis Kit (R&D Systems, Gaithersburg, USA) [22].

### Enzyme-linked immunosorbent assay (ELISA)

The inflammatory situation of 16HBE cells was identified by the secretion of TNF-α, IL-6, and IL-1β. The concentration of inflammatory factors in cell supernatants was measured by the ELISA kits (CUSABIO, Wuhan, China) [23].

### Luciferase activity assay

The regions of OIP5-AS1 and IL-13 containing targeting sites were amplified by PCR, namely the wide type (WT) of OIP5-AS1 and IL-13. The mutation (MUT) of OIP5-AS1 and IL-13 were synthesized by GenePharma (Shanghai, China). These obtained sequences were cloned into psiCHECK2 vectors respectively. Then, miR-NC, miR-410-3p mimics, and miR-410-3p inhibitors were transfected into cells with four different carriers respectively. After 48 h of transfection, we followed the instructions of the double luciferase reporter gene detection kit to detect luciferase activity (YEASEN, Shanghai, China) [24]. The Synergy H4 multifunctional microplate reader was used to read the fluorescence value and calculate the relative fluorescence activity.

### Western blot

The total protein was isolated from 16HBE cells using pre-cooled RIPA lysis buffer (Solarbio, Beijing, China) and the concentration was detected by BCA reagent (Yise, Shanghai, China). The experiments of Western blot were conducted following a previous study [25]. The protein (20 μg) was separated by 10% SDS-PAGE after being degenerated and were then electro-transferred to 0.22 µm PVDF membranes (Millipore, Billerica, MA). The membranes were blocked with 5% BSA solution for 2 h at room temperature and then incubated with primary antibody (ab106732; Abcam) at 4°C overnight. Membranes were washed with TBST and horseradish peroxidase labeled secondary antibody was added to the membranes for one hour. The ECL luminescent solution (Glpbio, Montclair, USA) was used to visualized protein bands. Grayscale values were analyzed with ImageJ and GAPDH was used as loading control.

### Statistical analysis

The results were drawn by SPSS and GraphPad, in which all measurement data were demonstrated as x ± s. Student t-test, one-way analysis of variance, and chi-square test were used to analyze the mean value of groups. Pearson variable linear test was used for correlations. The receiver operating characteristic (ROC) curve was used to predict the clinical significance of OIP5-AS1. *P* < 0.05 indicated that the comparison was significant.

## Results

This investigation was aimed to study the clinical significance and possible mechanism of OIP5-AS1 in COPD. A hypothesis was conducted that OIP5-AS1 might regulate severity of COPD via controlling specific downstream target. To verify this hypothesis, we confirmed the expression of OIP5-AS1 in COPD patients and analyzed the association between OIP5-AS1 and COPD. Besides, in vitro assays were performed to reveal the potential mechanism of OIP5-AS1 in COPD.

### Clinical features of the study population

Demographic data of patients are obtained from medical records. The mean age of 62 COPD cases was 59.34 ± 8.49 years old. There were 55 nonsmokers in our study with a mean age of 57.84 ± 8.40 years old and 59 smokers with a mean age of 59.83 ± 8.45 years old. No significant differences in age, gender, and body-mass index (BMI) were found among the nonsmoker group, smoke group, and COPD group (Table 1, *P* > 0.05). Besides, the smoking history, occupational dust exposure, FEV1, and FEV1/FVC were also recorded in this study. The mean smoking history and number of occupational dust exposure among the nonsmoker group, smoke group, and COPD group showed no difference (Table 1, *P* > 0.05). In addition, a significant reduction in FEV1 and FEV1/FVC was found in the COPD group (Table 1, *P* < 0.001).

**Table 1.** Characters of subjects in the current study

Characteristics	Nonsmoker (n = 55)	Smoker (n = 59)	COPD (n = 62)	*P* value
Age (years)	57.84 ± 8.40	59.83 ± 8.45	59.34 ± 8.49	0.427
Gender (male/female)	37/18	43/16	51/11	0.169
BMI (kg/m^2^)	22.68 ± 2.99	22.24 ± 2.32	22.25 ± 2.33	0.647
Smoking (pack-years)	-	40.68 ± 7.86	42.16 ± 9.28	0.196
Occupational dust exposure (No/Yes)	49/6	50/9	55/7	0.734
FEV1 (%)	94.70 ± 6.22	92.71 ± 6.68	57.23 ± 13.49	<0.001
FEV1/FVC (%)	82.97 ± 4.90	83.92 ± 4.36	55.66 ± 8.04	<0.001

### Expression and diagnostic accuracy of OIP5-AS1 for COPD patients

**Figure 1.**
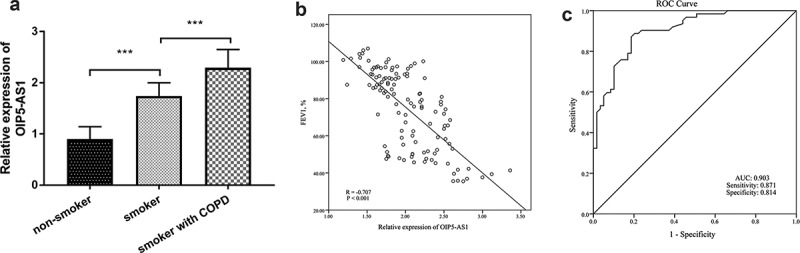
(a) The increased levels of OIP5-AS1 in smokers and smokers with COPD. (b) The relationship between OIP5-AS1 and FEV1. (c) The diagnostic roles of OIP5-AS1. ***P < 0.001

The levels of OIP5-AS1 in different groups were confirmed by the qRT-PCR system. The finding showed that the expression of OIP5-AS1 was elevated in smokers when relative to nonsmokers (Figure 1a, *p* < 0.001). In addition, OIP5-AS1 was at a high level in smokers with COPD (Figure 1a, *p* < 0.001). Besides, the association between the expression of OIP5-AS1 and FEV1 in all smokers was corroborated by Pearson analysis. As shown in Figure 1b, there was a negative connection from OIP5-AS1 to FEV1 (R = −0.707, *P* < 0.001). Furthermore, the prognostic value of OIP5-AS1 on differentiating COPD patients with smoking history from smokers was unveiled by the area under the curve (AUC). The AUC of 0.903 indicated that OIP5-AS1 might be an indicator of distinguishing COPD patients (Figure 1(c)). The sensitivity was 0.871 and the specificity was 0.814 when the cutoff value was 0.685 (Figure 1(c)).

### The impacts of OIP5-AS1 on CSE-treatment cells

**Figure 2.**
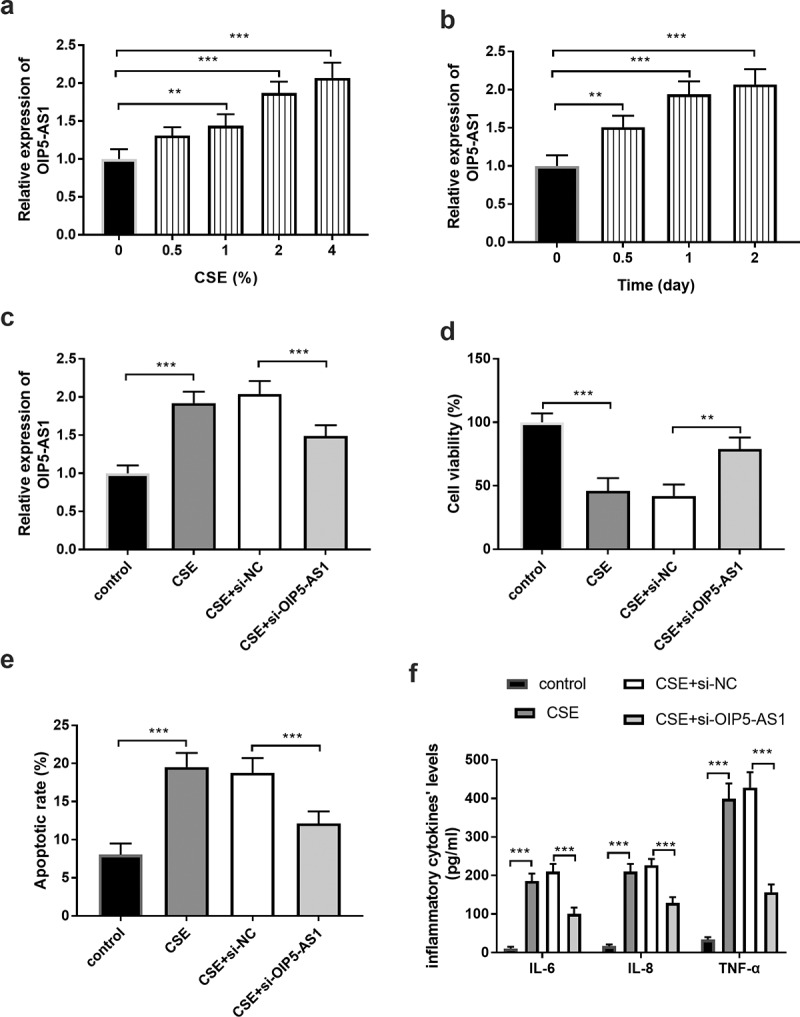
(a-b) The levels of OIP5-AS1 in cells treated with different concentrations of CSE at different times. (c) The expression of OIP5-AS1 in 16HBE cells. (d-f) The impacts of OIP5-AS1 on CSE- triggered cells. **P < 0.01, ***P < 0.001

The cell model of COPD was established by the treatment on 16HBE using CSE. As exhibited in (Figure 2(a)), the relative levels of OIP5-AS1 in 16HBE cells were raised accompanied by the increasing concentration of CSE (*P* < 0.01). Besides, along with the increased treating time, the levels of OIP5-AS1 were enhanced in 16HBE cells (Figure 2(b), *p* < 0.01). Combining the findings of concentration and time, the cell models were constructed by treating 16HBE cells with 2% CSE for 24 hours.

Additionally, the levels of OIP5-AS1 were regulated by the transfection of si-OIP5-AS1. The relative levels of OIP5-AS1 were higher in the CSE group than in the control cells, while si- OIP5-AS1 reversed this trend (Figure 2(c), *p* < 0.001). The outcome of cell viability provided that CSE treatment inhibited the cell viability, whereas, the decreased OIP5-AS1 levels suppressed the restricted cell viability (Figure 2(d), *p* < 0.001). The apoptotic rate of cells in the CSE group was prominently elevated, while downregulation of OIP5-AS1 alleviated the aberrant cell apoptosis (Figure 2(e), *p* < 0.001). The management of CSE contributed to the increased levels of inflammatory factors and the anomalous inflammatory situation was moderated by the reduced OIP5-AS1 expression (figure 2(f), *p* < 0.001).

### OIP5-AS1 was a sponge of miR-410-3p

**Figure 3.**
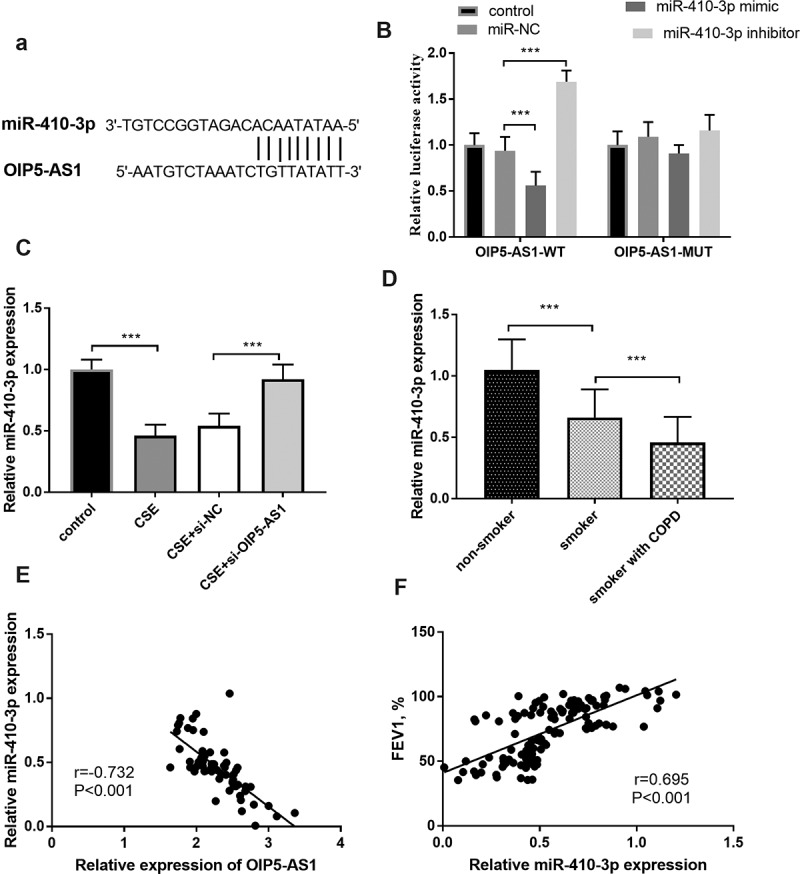
(a) The complementary sites between miR-410-3p and OIP5-AS1. (b) The results of the luciferase assay. (c) The alteration of miR-410-3p in 16HBE cells. (d) The declined levels of OIP5-AS1 in smokers and smokers with COPD. (e) The connection between miR-410-3p and OIP5-AS1. (e) The correlation between miR-410-3p and FEV1. **P < 0.01, ***P < 0.001

The putative target sites between miR-410-3p and OIP5-AS1 were exhibited in (Figure 3(a)), which elucidated the possible relationship between OIP5-AS1 and miR-410-3p. A further demonstration was provided by the luciferase results, which discovered that the miR-410-3p mimics inhibited the luciferase activity and interference of miR-410-3p promoted the luciferase activity in the OIP5-AS1-WT group (Figure 3(b), *p* < 0.001). Moreover, the relative miR-410-3p expression in the CSE-steered cells was meliorated relative to those in the control group, while the lowly expressed OIP5-AS1 mitigated the reduction of miR-410-3p expression (Figure 3(c), *p* < 0.001).

### Lessened miR-410-3p expression in COPD patients

In participants, a diminished level of miR-410-3p was observed in the smokers relative to the nonsmokers (Figure 3(d), *p* < 0.001). Besides, the expression of miR-410-3p was declined in smokers with COPD relative to the smokers (Figure 3(d), *p* < 0.001). The Pearson correlation validated the close association between miR-410-3p and OIP5-AS1 in smokers with COPD (Figure 3(e), r = −0.732, *P* < 0.001). More importantly, in all smokers, the relative miR-410-3p expression was positively linked to the levels of FEV1 (Figure 3(f), r = 0.695, *P* < 0.001).

### Influence of miR-410-3p on CSE-triggered cells

**Figure 4.**
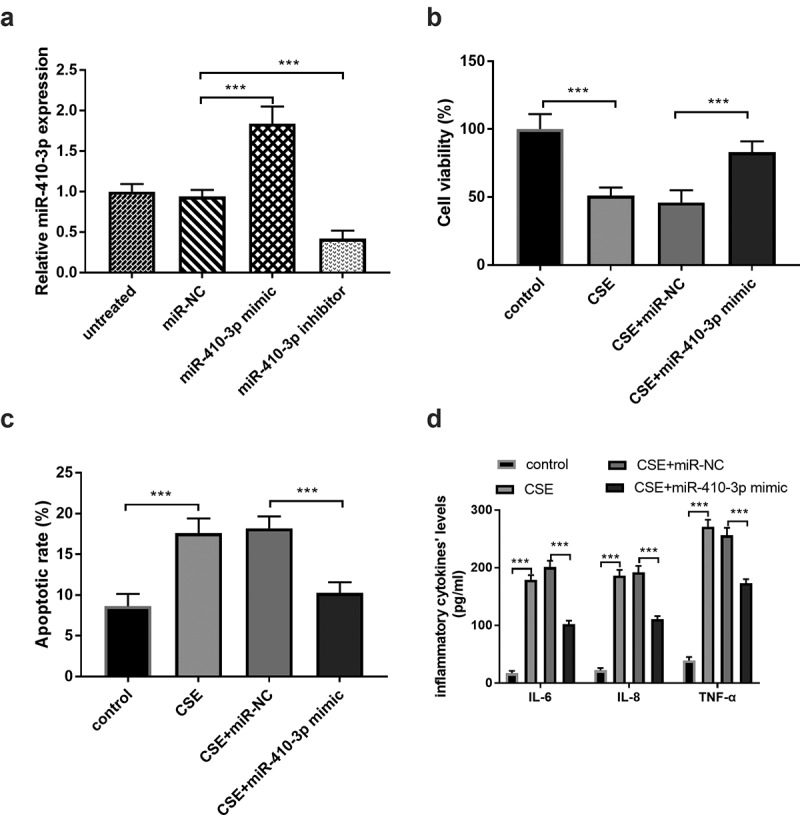
(a) The artificial regulation of miR-410-3p expression. (b-c) The impacts of miR-410-3p on CSE- stimulated cells. ***P < 0.001

The effects of miR-410-3p in the 16HBE cells under the CSE situation were evaluated in this report. The miR-410-3p expression was conspicuously increased in the miR-410-3p mimic group and decreased in the cells transfected with miR-410-3p inhibitor (Figure 4(a), *p* < 0.001). The ascended miR-410-3p expression reversed the influence of CSE on cell viability and apoptosis (Figure 4(b-C), *P* < 0.001), suggesting the beneficial roles of miR-410-3p on the CSE-injured cells. Furthermore, the inflammatory response in the CSE-engendered cells was meliorated by the abundance of miR-410-3p (Figure 4(d), *p* < 0.001).

### MiR-410-3p mediated the effects of OIP5-AS1

**Figure 5.**
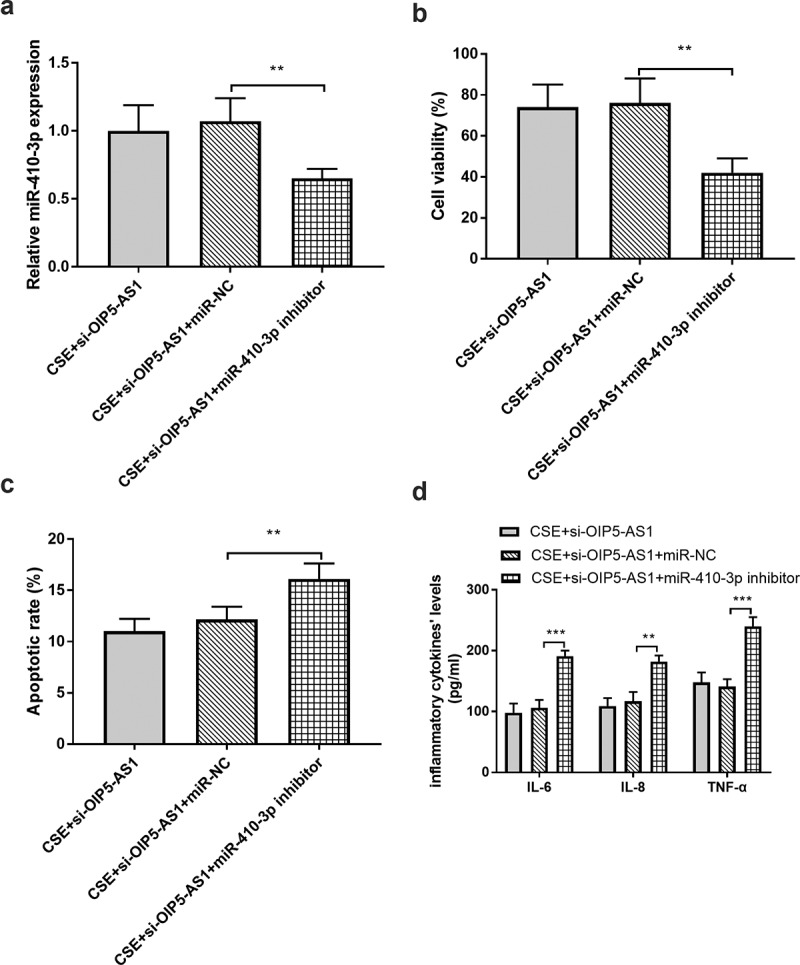
(a) miR-410-3p The mediated effects of miR-410-3p on **A** miR-410-3p expression, (b) cell viability, (c) apoptotic rate, and (d) inflammation. **P < 0.01, ***P < 0.001

The sequences of si-OIP5-AS1 and miR-410-3p inhibitor were co-transfected in 16HBE cells to regulate the expression of miR-410-3p. As shown in (Figure 5(a)), miR-410-3p expression was reduced in the CSE + si-OIP5-AS1 + miR-410-3p inhibitor group when compared to the CSE + si-OIP5-AS1 inhibitor group (*P* < 0.01). The assay on cell viability clarified that in the cells co-transfected with si-OIP5-AS1 and miR-410-3p inhibitor, the cell viability was repressed relative to the CSE + si-OIP5-AS1 inhibitor group (Figure 5(b), *p* < 0.01). Besides, the reduced miR-410-3p levels changed the impacts of si-OIP5-AS1 on apoptotic rate and inflammation (Figure 5(c-d), *P* < 0.01).

### IL-13 served as a target gene of miR-410-3p

**Figure 6.**
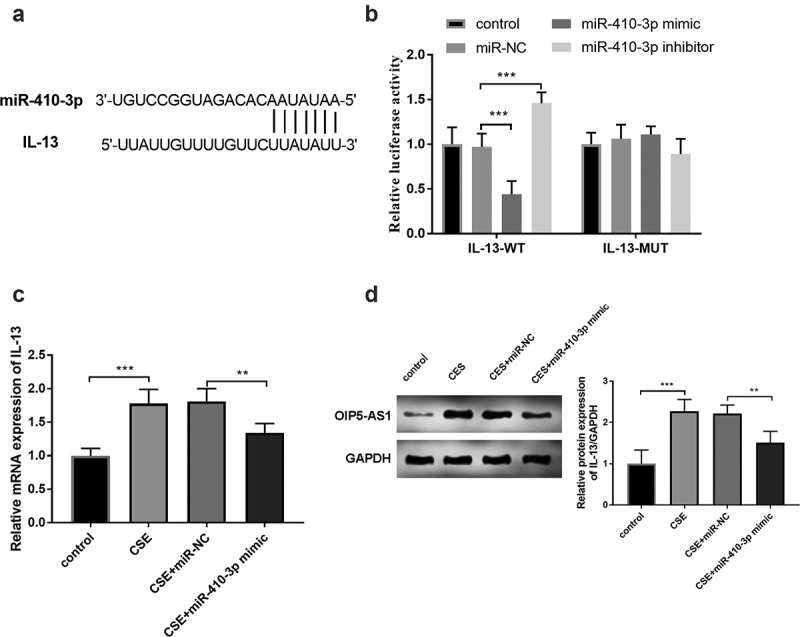
(a) The complementary bases between miR-410-3p and IL-13. (b) The exhibition of the luciferase report analysis. (c) The alternation of IL-13 mRNA expression in 16HBE cells. (d) The protein expression of IL-13 in 16HBE cells. **P < 0.01, ***P < 0.001

The complementary bases between miR-410-3p and IL-3 were shown in (Figure 6(a)). This target association between miR-410-3p and IL-3 was examined by the results of the luciferase report. In (Figure 6(b)), the increased expression of miR-410-3p repressed the luciferase activity and interference of miR-410-3p improved the luciferase activity (*P* < 0.001). The mRNA expression of IL-13 was increased in the CSE-stimulated cells and reversed by the enhanced miR-410-3p expression (Figure 6(c), *p* < 0.01). Furthermore, the relative protein expression of OIP5-AS1 was raised in the CSE models and repressed in the CSE+miR-410-3p mimic group (Figure 6(d), *p* < 0.01).

**Figure 7.**
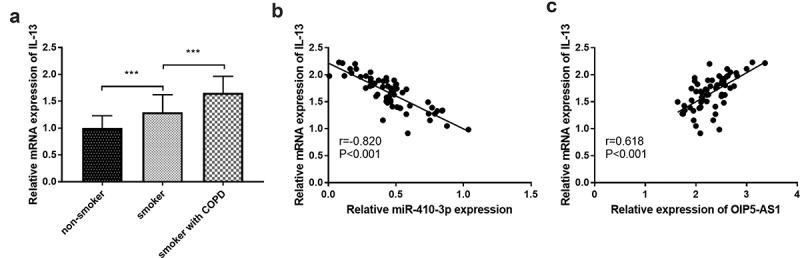
(a) The declined mRNA levels of IL-13 in smokers and smokers with COPD. (b) The target relation between miR-410-3p and IL-13. (c) The association between IL-13 and OIP5-AS1. ***P < 0.001

In clinical experiments, IL-13 was at high levels both in smokers and smokers with COPD (Figure 7(a), *p* < 0.001). In all smokers with COPD, close correlations were both found between IL-13 and miR-410-3p (Figure 7(b), r = −0.820, *P* < 0.001) together with IL-13 and OIP5-AS1 (Figure 7(c), r = 0.618, *P* < 0.001).

## Discussion

The more common complication in COPD is pulmonary hypertension, which will exert a serious influence on the prognosis and life quality of patients, and increase the risk of hospitalization and prolong the duration of hospitalization [26,27]. Clinically, it is considered that the cause of pulmonary hypertension in patients with COPD is not only related to hypoxia, but also other causes. In recent years, the important role of chronic pulmonary inflammatory situations in the occurrence of pulmonary hypertension has been studied [28]. When COPD occurs in patients with complications, it will lead to heart failure, cor pulmonale, and so on [29]. Thus, such conditions necessitate the investigation of the realms of molecular mechanisms in COPD.

Involvements of lncRNAs and the physiological and pathological diseases of the lung were reported, such as asthma, COPD, and pulmonary fibrosis [30]. The expression of MALAT1 is enhanced in patients with asthma and it participates in the regulation of inflammation by sponging miR-155 [31]. The underexpression of HOXA- AS2 is found in patients with COPD and it exerts an essential impact on cell proliferation by interfering with Notch1 expression [32]. Hundreds of studies have confirmed the anomalous expression of lncRNA in lung tissue of COPD, and some lncRNA showed obvious and specific expression, which will help to find early biomarkers and drug treatment targets related to COPD. In an investigation about chronic respiratory diseases, the expression of OIP5-AS1 is elevated in COPD patients compared with patients with asthma [33]. The current study indicated that the levels of OIP5-AS1 were elevated in smokers and smokers with COPD, which provided that OIP5-AS1 may be used as an early biomarker of COPD occurrence and development. Besides, we found in all smokers, the expression of OIP5-AS1 was relative to the FEV1, suggesting the close association between OIP5-AS1 and COPD. More importantly, the diagnostic value of OIP5-AS1 was indicated by the ROC curve and the finding showed that OIP5-AS1 could discriminate smokers with COPD patients from smokers.

To provide theoretical guidance for the early diagnosis of COPD, the mechanism of the effect of OIP5-AS1 on the development of COPD was discussed. The expression of OIP5-AS1 is raised in the CSE-treated human bronchial epithelial cells which is consistent with the levels of patients with COPD. Furthermore, the knockdown of OIP5-AS1 in the CSE-engendered 16HBE cells reversed the influence of CSE on cell viability and apoptosis, indicating the beneficial roles of silenced OIP5-AS1. Besides, the inflammation in 16HBE cells was activated by the CSE and attenuated by the intervention of OIP5-AS1. All these findings manifested the reduced OIP5-AS1 expression could abrogate the impacts of CSE partially. More and more studies have shown that OIP5-AS1 has great potential value in lung diseases. In a cell model of bronchial asthma, the elimination of OIP5-AS1 expression inhibits the inflammatory situation and apoptosis [14]. In lung cancer, the overexpression of OIP5-AS1 is found in the tissues of patients and it can improve cell proliferation [34].

In addition, lncRNA can inhibit the expression of miRNA to regulate gene expression and regulate physiological and pathological processes. In our investigation, miR-410-3p is confirmed to be a target of OIP5-AS1. Besides, the expression level of miRNAs could be regulated by silencing or overexpressing lncRNA, suggesting that miRNA might play a significant role in the progression of COPD. The relative levels of miR-410-3p in CSE-steered cells were reduced, while the absence of OIP5-AS1 reversed the restricted miR-410-3p expression, validating the target relationship between OIP5-AS1 and miR-410-3p. A previous outcome elucidates that miR-410-3p participates in the EMT and radio resistance by activating PTEN/PI3K/mTOR pathway in non-small cell lung cancer [35]. In an ovalbumin‑managed murine model of asthma, the expression of miR-410-3p was diminished and it might be a target of treating allergic asthma [36]. In the current report, the relative expression of miR-410-3p was lessened in smokers and smokers with COPD. Moreover, the relative levels of miR-410-3p were associated with the expression of OIP5-AS1 in smokers. The expression of miR-410-3p was relative to the FEV1, indicating the miR-410-3p expression might be influenced by COPD. In vitro, many kinds of research about the function of miR-410-3p on cell viability, growth, and apoptosis have been performed. In rheumatoid arthritis, the overexpression of miR-410-3p restricted proliferation, promoted apoptosis by inhibiting YY1 [37]. Besides, an abundance of miR-410-3p levels protects the neuronal cell against 6-OHDA on viability, apoptosis, and reactive oxygen species secretion [38]. In this investigation, the enhancement of miR-410-3p partially reversed the effects of CSE on cell viability, death, and inflammatory progression. What’s more, miR-410-3p also mediated the function of OIP5-AS1 on viable cells, apoptotic cells, and inflammatory situations, which provided further confirmation of the connection between miR-410-3p and OIP5-AS1.

In the current publication, IL-13 might be a downstream gene of miR-410-3p by the elucidation of luciferase report, which provided that the enforced miR-410-3p levels mitigated the luciferase activity and the reduced miR-410-3p improved the luciferase activity. Besides, the CSE might contribute to the high expression of IL-13, while the enhanced miR-410-3p attenuated the variation of IL-13 expression. The expression and mechanism of IL-13 have been examined by other experts. In an article of 2019, the concentration of IL-13 is raised in COPD and other lung disorders [39]. The expression of IL-13 in CD8 + T cells of COPD patients is obviously elevated compared to the smokers without lung dysfunction [40]. In asthma, intranasal miR-140-3p inhibits the inflammatory responses by suppressing the levels of eosinophils and IL-13 [36]. We also identified IL-13 as at a high level in smokers with COPD and smokers. Besides, the mRNA expression of IL-13 was relative to the expression of miR-410-3p and OIP5-AS1 in smokers with COPD.

## Conclusion

In summary, the expression of OIP5-AS1 was enhanced in smokers and smokers with COPD, and the ascended levels of OIP5-AS1 were associated with the low levels of FEV1. OIP5-AS1 showed promising predictive significance in differentiating smokers with COPD from smokers. MiR-410-3p was at low levels and IL-13 was at high levels in smokers and smokers with COPD. Knockdown of OIP5-AS1 promoting the recovery of cells from CSE damage by motivating cell viability, moderating cell apoptosis, and meliorating inflammatory situation via miR-410-3p and IL-13.

## References

[1] Lareau SC, Fahy B, Meek P, et al. Chronic obstructive pulmonary disease (COPD). Am J Respir Crit Care Med. 2019 Jan 1;199(1):P1–p2. 10.1164/rccm.1991P1 30592446

[2] Kim SH, Hong JH, Yang WK, et al. Herbal combinational medication of Glycyrrhiza glabra, Agastache rugosa Containing Glycyrrhizic Acid, Tilianin inhibits neutrophilic lung inflammation by affecting CXCL2, Interleukin-17/STAT3 signal pathways in a murine model of COPD. Nutrients. 2020 Mar 27;12:4. 10.3390/nu12040926 PMC7231088 32230838

[3] Esquinas C, Janciauskiene S, Gonzalo R, et al. Gene and miRNA expression profiles in PBMCs from patients with severe and mild emphysema and PiZZ alpha1-antitrypsin deficiency. Int J Chron Obstruct Pulmon Dis. 2017;12:3381–3390. 29238183 10.2147/COPD.S145445 PMC5713702

[4] Labaki WW, Rosenberg SR. Chronic obstructive pulmonary disease. Ann Intern Med. 2020 Aug 4;173(3):Itc17–itc32. 32745458 10.7326/AITC202008040

[5] Qi X, Chen H, Fu B, et al. LncRNAs NR-026690 and ENST00000447867 are upregulated in CD4(+) T cells in patients with acute exacerbation of COPD. Int J Chron Obstruct Pulmon Dis. 2019;14:699–711. 30988604 10.2147/COPD.S191815 PMC6440447

[6] Rabe KF, Watz H. Chronic obstructive pulmonary disease. Lancet. 2017 May 13;389(10082):1931–1940. 28513453 10.1016/S0140-6736(17)31222-9

[7] Celli BR, Wedzicha JA, Drazen JM. Update on clinical aspects of chronic obstructive pulmonary disease. N Engl J Med. 2019 Sep 26;381(13):1257–1266. 31553837 10.1056/NEJMra1900500

[8] Hattab Y, Alhassan S, Balaan M, et al. Chronic obstructive pulmonary disease. Crit Care Nurs Q. 2016 Apr-Jun;39(2):124–130. 26919673 10.1097/CNQ.0000000000000105

[9] Poulet C, Njock MS, Moermans C, et al. Exosomal long non-coding RNAs in lung diseases. Int J Mol Sci. 2020 May 19;21:10. 10.3390/ijms21103580 PMC7279016 32438606

[10] Song J, Wang Q, LncRNA ZL. MIR155HG contributes to smoke-related chronic obstructive pulmonary disease by targeting miR-128-5p/BRD4 axis. Biosci Rep. 2020 Mar 27;40(3). DOI:10.1042/BSR20192567. PMC7070147 32129458

[11] Hu TJ, Huang HB, Shen HB, et al. Role of long non-coding RNA MALAT1 in chronic obstructive pulmonary disease. Exp Ther Med. 2020 Sep;20(3):2691–2697. 32765763 10.3892/etm.2020.8996 PMC7401856

[12] Niu X, Pu S, Ling C, et al. lncRNA Oip5-as1 attenuates myocardial ischaemia/reperfusion injury by sponging miR-29a to activate the SIRT1/AMPK/PGC1α pathway. Cell Prolif. 2020 Jun;53(6):e12818. 32468629 10.1111/cpr.12818 PMC7309946

[13] Deng J, Deng H, Liu C, et al. Long non-coding RNA OIP5-AS1 functions as an oncogene in lung adenocarcinoma through targeting miR-448/Bcl-2. Biomed Pharmacothe. 2018 Feb;98:102–110. 10.1016/j.biopha.2017.12.031 29247949

[14] Cai XJ, Huang LH, Zhu YK, et al. LncRNA OIP5‑AS1 aggravates house dust mite‑induced inflammatory responses in human bronchial epithelial cells via the miR‑143‑3p/HMGB1 axis. Mol Med Rep. 2020 Dec;22(6):4509–4518. 33174035 10.3892/mmr.2020.11536 PMC7646745

[15] Vogelmeier CF, Criner GJ, Martinez FJ, et al. Global strategy for the diagnosis, management, and prevention of chronic obstructive lung disease 2017 report. GOLD executive summary. Am J Respir Crit Care Med. 2017 Mar 1;195(5):557–582. 28128970 10.1164/rccm.201701-0218PP

[16] Liu P, Zhang H, Zeng H, et al. LncRNA CASC2 is involved in the development of chronic obstructive pulmonary disease via targeting miR-18a-5p/IGF1 axis. Ther Adv Respir Dis. 2021 Jan-Dec;15:17534666211028072. 34266334 10.1177/17534666211028072 PMC8290508

[17] Zhou F, Cao C, Chai H, et al. Circ-HACE1 aggravates cigarette smoke extract-induced injury in human bronchial epithelial cells via regulating toll-like receptor 4 by sponging miR-485-3p. Int J Chron Obstruct Pulmon Dis. 2021;16:1535–1547. 34103911 10.2147/COPD.S304859 PMC8179752

[18] Zhao S, Lin C, Yang T, et al. Expression of long non-coding RNA LUCAT1 in patients with chronic obstructive pulmonary disease and its potential functions in regulating cigarette smoke extract-induced 16HBE cell proliferation and apoptosis. J Clin Lab Anal. 2021 Jul;35(7):e23823. 34125980 10.1002/jcla.23823 PMC8274995

[19] Yang P, Han J, Li S, et al. miR-128-3p inhibits apoptosis and inflammation in LPS-induced sepsis by targeting TGFBR2. Open Med (Wars). 2021;16(1):274–283. 33623823 10.1515/med-2021-0222 PMC7885300

[20] Xiaoling G, Shuaibin L, Kailu L. MicroRNA-19b-3p promotes cell proliferation and osteogenic differentiation of BMSCs by interacting with lncRNA H19. BMC Med Genet. 2020 Jan 9;21(1):11. 31918667 10.1186/s12881-020-0948-y PMC6953218

[21] Liu K, Zhou S, Liu J, et al. Silibinin attenuates high-fat diet-induced renal fibrosis of diabetic nephropathy. Drug Des Devel Ther. 2019;13:3117–3126. 10.2147/DDDT.S209981 PMC6718242 31695328

[22] Nylund P, Atienza Párraga A, Haglöf J, et al. A distinct metabolic response characterizes sensitivity to EZH2 inhibition in multiple myeloma. Cell Death Dis. 2021 Feb 12;12(2):167. 33579905 10.1038/s41419-021-03447-8 PMC7881125

[23] Liu J, Jiang M, Deng S, et al. miR-93-5p-containing exosomes treatment attenuates acute myocardial infarction-induced myocardial damage. Mol Ther Nucleic Acids. 2018 Jun 1;11:103–115. 29858047 10.1016/j.omtn.2018.01.010 PMC5852413

[24] Ma J, Zhang X, Zhang H, et al. lncRNA MEG3 suppresses the progression of ankylosis spondylitis by regulating the Let-7i/SOST axis. Front Mol Biosci. 2020;7:173. 32793634 10.3389/fmolb.2020.00173 PMC7393269

[25] Zhang Q, Wu G, Guo S, et al. Effects of tristetraprolin on doxorubicin (Adriamycin)-induced experimental kidney injury through inhibiting IL-13/STAT6 signal pathway. Am J Transl Res. 2020;12(4):1203–1221. 32355536 PMC7191163

[26] Jin G, Chen Z, Zhang J, et al. Association of brain natriuretic peptide gene polymorphisms with chronic obstructive pulmonary disease complicated with pulmonary hypertension and its mechanism. Biosci Rep. 2018 Oct 31;38(5). DOI:10.1042/BSR20180905. PMC6167498 30217946

[27] Zhang Y, Lin P, Hong C, et al. Serum cytokine profiles in patients with chronic obstructive pulmonary disease associated pulmonary hypertension identified using protein array. Cytokine. 2018 Nov;111:342–349. 30273784 10.1016/j.cyto.2018.09.005

[28] Pilato E, Manzo R, Comentale G. Pulmonary embolism and Sars-Cov-2 infection: a new indication for surgical pulmonary endarterectomy? Heart Lung. 2020 Jul-Aug;49(4):352. 32425273 10.1016/j.hrtlng.2020.05.007 PMC7231736

[29] Cau R, Bassareo PP, Mannelli L, et al. Imaging in COVID-19-related myocardial injury. Int J Cardiovasc Imaging. 2021 Apr;37(4):1349–1360. 33211242 10.1007/s10554-020-02089-9 PMC7676417

[30] Li Y, Yin Z, Fan J, et al. The roles of exosomal miRNAs and lncRNAs in lung diseases. Signal Transduct Target Ther. 2019;4:47. 31728212 10.1038/s41392-019-0080-7 PMC6851157

[31] Liang Z, Tang F. The potency of lncRNA MALAT1/miR-155/CTLA4 axis in altering Th1/Th2 balance of asthma. Biosci Rep. 2020 Feb 28;40(2). DOI:10.1042/BSR20190397. PMC7024843 31909418

[32] Zhou AY, Zhao YY, Zhou ZJ, et al. Microarray analysis of long non-coding RNAs in lung tissues of patients with COPD and HOXA-AS2 promotes HPMECs proliferation via notch1. Int J Chron Obstruct Pulmon Dis. 2020;15:2449–2460. 33116460 10.2147/COPD.S259601 PMC7555270

[33] Gál Z, Gézsi A, Semsei ÁF, et al. Investigation of circulating lncRNAs as potential biomarkers in chronic respiratory diseases. J Transl Med. 2020 Nov 10;18(1):422. 33168013 10.1186/s12967-020-02581-9 PMC7653503

[34] Wang M, Sun X, Yang Y, et al. Long non-coding RNA OIP5-AS1 promotes proliferation of lung cancer cells and leads to poor prognosis by targeting miR-378a-3p. Thorac Cancer. 2018 Aug;9(8):939–949. 29897167 10.1111/1759-7714.12767 PMC6068457

[35] Yuan Y, Liao H, Pu Q, et al. miR-410 induces both epithelial-mesenchymal transition and radioresistance through activation of the PI3K/mTOR pathway in non-small cell lung cancer. Signal Transduct Target Ther. 2020 Jun 12;5(1):85. 32528035 10.1038/s41392-020-0182-2 PMC7290026

[36] Jin R, Hu S, Liu X, et al. Intranasal instillation of miR‑410 targeting IL‑4/IL‑13 attenuates airway inflammation in OVA‑induced asthmatic mice. Mol Med Rep. 2019 Feb;19(2):895–900. 30535486 10.3892/mmr.2018.9703 PMC6323201

[37] Wang Y, Jiao T, Fu W, et al. miR-410-3p regulates proliferation and apoptosis of fibroblast-like synoviocytes by targeting YY1 in rheumatoid arthritis. Biomed Pharmacothe. 2019 Nov;119:109426. 10.1016/j.biopha.2019.109426 31505424

[38] Ge H, Yan Z, Zhu H, et al. MiR-410 exerts neuroprotective effects in a cellular model of Parkinson’s disease induced by 6-hydroxydopamine via inhibiting the PTEN/AKT/mTOR signaling pathway. Exp Mol Pathol. 2019 Aug;109:16–24. 31067440 10.1016/j.yexmp.2019.05.002

[39] Vázquez Y, González L, Noguera L, et al. Cytokines in the respiratory airway as biomarkers of severity and prognosis for respiratory syncytial virus infection: an update. Front Immunol. 2019;10:1154. 31214165 10.3389/fimmu.2019.01154 PMC6557983

[40] Wang YQ, Liao Q, Tang SH, et al. Gubenzhike recipe ameliorates respiratory mucosal immunity in mice with chronic obstructive pulmonary disease through upregulation of the γδT lymphocytes and KGF levels. Evid Based Complement Alternat Med. 2020;2020:3056797. 32280354 10.1155/2020/3056797 PMC7128036

